# Manipulation of nitric oxide levels with a modified hydroxyethyl starch molecule

**DOI:** 10.1186/cc11753

**Published:** 2012-11-14

**Authors:** C Lupp, S Baasner, D Heckmann, C Ince, F Nocken, M Schimmel, M Westphal

**Affiliations:** 1 Fresenius Kabi Deutschland GmbH, Bad Homburg, Germany; 2 Fresenius Kabi Deutschland GmbH, Friedberg, Germany; 3 Academic Medical Center, Amsterdam, the Netherlands

## Background

As mortality of patients with severe sepsis and septic shock is still inappropriately high [1], innovative therapeutic approaches are urgently needed. In the presence of hypovolemia, fluid therapy is typically initiated to compensate for intravascular volume deficits [2]. However, administration of fluids may not necessarily correct a disturbed blood flow on the microcirculatory level [3]. Microcirculatory failure during sepsis is, at least in part, caused by pathological nitric oxide (NO) levels [4,5]. To achieve an optimal NO availability, approaches including NO donors or inhibitors may be useful. The aim of the present study was to assess the ability of our test substance S-nitrosothiol-HES (S-NO-HES) to act as NO donor and exert a pharmacological activity.

## Methods

The investigated test substance S-NO-HES is a novel molecule consisting of NO coupled to a thiolated derivative of hydroxyethyl starch (HES). The ability of S-NO-HES to release NO was demonstrated. Furthermore, the effect of S-NO-HES on myocardial function was studied in isolated Langendorff-perfused hearts from guinea pigs and compared with that of the reference substance sodium nitroprusside. The thiolated HES derivative (SH-HES) served as negative control. In addition, isolated aortic rings from rats were pre-contracted by phenylephrine. After defined incubation periods with reference, test, or control items, the NO-induced relaxation was determined. S-nitrosoglutathione served as reference compound, HES and in one experiment also SH-HES as control substances. At the end of the 180-minute experiment, papaverine was applied in order to completely relax the aortic rings and to define the 100% relaxation level.

## Results

S-NO-HES significantly increased the heart rate of Langendorff-perfused guinea pig hearts and additionally reduced both the QT interval and QTc-B values. In addition, S-NO-HES exerted a significant vasodilatory effect on phenylephrine pre-contracted rat aortic rings that was dose dependent. The effect was not only observed under light, which is known to trigger NO release from S-nitroso compounds, but also under exclusion of light and therefore more physiological conditions.

## Conclusion

We demonstrated for the first time that the S-NO-HES molecule released NO and exhibited corresponding pharmacological properties. In future experiments, the effectiveness of S-NO-HES to substitute NO deficiency under septic conditions has to be studied.
